# Sustainable Farming: Nanofiber from the Pupunha Heart of Palm Sheath (*Bactris gasipaes*)-Enhanced Diets for Growing Rabbits and Their Health Impacts

**DOI:** 10.3390/vetsci12030263

**Published:** 2025-03-12

**Authors:** Geovane Rosa de Oliveira, Carla de Andrade, Celina Tie Nishimori Duque, Antonio Diego Brandão Melo, Cristina Santos Sotomaior, Washington Luiz Esteves Magalhães, Saulo Henrique Weber, Fernando Bittencourt Luciano, Leandro Batista Costa

**Affiliations:** 1 Graduate Program in Animal Science, School of Medicine and Life Sciences, Pontifícia Universidade Católica do Paraná (PUCPR), Curitiba 80215-901, Paraná, Brazil; geovane.oliveira@pucpr.edu.br (G.R.d.O.); carlazoobr@yahoo.com.br (C.d.A.); celina.duque@pucpr.br (C.T.N.D.); diegobmelo@hotmail.com (A.D.B.M.); cristina.sotomaior@pucpr.br (C.S.S.); saulo.weber@pucpr.br (S.H.W.); fernando.luciano@pucpr.br (F.B.L.); 2 Monohub—Research Group for Monogastric Animals, Pontifícia Universidade Católica do Paraná (PUCPR), Curitiba 80215-901, Paraná, Brazil; 3 Embrapa Forest and Nanotechnology, Colombo 83411-000, Paraná, Brazil; washington.magalhaes@embrapa.br

**Keywords:** animal health, co-products, fiber, nanostructure, nutrition, sustainable

## Abstract

The use of nanofibers in farm animal diets can enhance nutrient absorption, minimize environmental problems, and generate a sustainable source of income. In this study, we investigated the effects of the partial inclusion of nanofibers produced from the pupunha heart of the palm sheath (nanopupunha) in the diet of growing New Zealand White rabbits on zootechnical performance, organ morphometry, digestive content pH, intestinal histology, biochemical and immunological parameters, and cecum microbiota. Twenty-four male and female New Zealand White rabbits were distributed into the control group fed a basal diet with 14% crude fiber and treatment groups with the basal diet supplemented with 3.5% or 10.5% of nanopupunha, according to their initial weight. After euthanasia on day 42, we analyzed the pH of the stomach contents, jejunum, and cecum, and the relative weights of the digestive tract, liver, kidneys, and spleen. Duodenal and jejunal samples were collected for structural and ultrastructural analyses of the intestinal villi. Additionally, blood samples were collected to analyze blood glucose, cholesterol, triglycerides, and immunological analysis (IgG and IgM), and digesta samples from the cecum were collected to count enterobacteria and lactic acid bacteria. The inclusion of dietary nanopupunha did not affect the zootechnical performance of animals, but resulted in a linear decrease in the relative weight of the stomach and a linear increase in the relative weight of the spleen. No significant differences were observed in the pH of the digestive tract. Nanopupunha inclusion also resulted in a linear increase in the crypt depth of the duodenum, total mucosal thickness, and total cholesterol levels in growing rabbits. Including 10.5% of nanopupunha added to the diet showed the best results in terms of the intestinal health of the growing rabbits.

## Simple Summary

The nanofibers derived from the sheath of the pupunha heart of palm (nanopupunha), when incorporated into the diet of growing rabbits, have shown the potential to enhance nutrient absorption, mitigate environmental challenges, and provide a sustainable source of income. Our study revealed significant effects of partially including dietary nanopupunha, such as a linear decrease in stomach relative weight and a linear increase in spleen relative weight. Furthermore, nanopupunha inclusion led to a linear increase in duodenal crypt depth, total mucosal thickness, and total cholesterol levels in growing rabbits. The best results for intestinal health were observed with a 10.5% inclusion of nanopupunha in the diet. These findings are crucial for advancing the understanding of nanostructure applications in animal nutrition and developing innovative ingredients to improve dietary strategies for animal production.

## 1. Introduction

From a global perspective, rabbit production offers significant advantages in terms of sustainability, as rabbit farming does not directly compete with human food resources [1]. Rabbit meat is recognized as a healthy and nutritious food due to its low fat content, high concentration of polyunsaturated fatty acids, proteins, and essential amino acids [2].

Rabbits have a unique physiological capacity to digest cellulose-rich foods, being able to convert up to 20% of the ingested protein into body weight gain [3]. Faostat (2024) [4] data highlights the role of rabbit meat in the global context, particularly in countries where access to low-cost, high-quality proteins is essential for food security. The digestive system of rabbits is particularly adapted, characterized by a relatively small stomach, a highly efficient small intestine for nutrient absorption, and a voluminous cecum where microbial fermentation occurs [5]. This adaptation allows fiber to play a critical role in intestinal health [6,7]. Balanced diets with appropriate fiber sources are fundamental to ensuring the health and performance of rabbits. Additionally, histological analysis of the digestive tract is essential to understand how different fiber sources affect intestinal function [5]. In particular, the duodenum and jejunum play critical roles in nutrient absorption. Histological studies of these regions can reveal structural changes, such as alterations in intestinal villi, which are directly related to diet and serve as important indicators of intestinal health.

The processing of the pupunha heart of palm sheath (*Bactris gasipeaes*) generates large amounts of waste (approximately 70% of its biomass) that can be used to obtain nanofibers. Cellulose nanofibers are produced from plant cell walls [8] and can be used for animal nutrition. Nanofibers possess specific physicochemical characteristics, such as long chains, increased surface area, and hydrogen bonds, which confer a high capacity for interaction with lipids and proteins [9,10]. These properties can facilitate enzymatic action during digestion and modulate beneficial microbial fermentation in the cecum.

Vegetable fibers are an abundant natural resource that can be used to feed several animal species; however, ref. [11] reported that the source, type, and fiber particle size can affect the development and health of the monogastric digestive tract. Moreover, digestible fiber and non-starch polysaccharides (NSPs) in the diet modulate the symbiotic microbiota in the cecum of rabbits, which ferments and converts the fiber into digestible components for the host [12,13]. In addition, with the increased industrial growth and demand for feed, it is necessary to develop new technologies and food resources to reduce production costs and ensure food security.

Incorporating nanofibers of the pupunha palm into a diet can bring advances in animal nutrition inherent to the nanoscale [14], given their biocompatibility, low weight, and hydroxyl groups present on their surface [15], which are capable of transporting nutrients and metabolites to cells. The greater surface area and smaller particle size of nanofibers [16] increase their application potential [17] and improve their incorporation into the diet and digestive capacity.

Some studies have highlighted the benefits of nanofibers [8,13,18], including digestion modulation, dietary fat utilization [19], antioxidant and adsorbent activity, and good acceptability by animals [20]. Studies have shown that nanofibers have potential anti-inflammatory effects on intestinal epithelial cells [21,22,23], reducing tissue injury caused by antinutritional factors. Other studies have shown the low cytotoxic potential of nanofibers of natural origin [16,24], enabling their use in animal feed to improve nutritional efficiency and contribute to a more sustainable production chain.

Although the effects of peach palm nanofibers on the development and health of the gastrointestinal tract of rabbits remain unexplored, it is already known that it is possible to use nanofibers in the rabbit diet. Additionally, evidence suggests that nanofibers can interact with intestinal epithelial cells, exerting an anti-inflammatory effect previously reported [25,26]. Absorption by intestinal cells can be enhanced by the presence of specific transporters in enterocytes, allowing greater nutrient assimilation throughout the digestive tract [27]. However, it is necessary to define the appropriate level of inclusion in the diet. Considering the beneficial effects of nanofibers, we hypothesized that nanopupunha could promote growth and development in rabbits. Our team published the first study evaluating the effects of nanofibers from soybean hulls and pupunha hearts of palm sheaths in the diet of growing rabbits [14] and observed that the pupunha peach palm improved the intestinal health of rabbits. However, in the present study, we investigated the optimal level of inclusion of nanofibers from the peach palm sheath on the zootechnical performance, organ morphometry, digestive content pH, intestinal histology, biochemical and immunological parameters, and cecum microbiota of growing rabbits.

## 2. Material and Methods

This experiment is a continuation of prior research examining the effects of dietary nanofibers on rabbit growth performance and health. Building on the methodologies established in the initial study, this experiment further investigates the impact of modified dietary compositions on specific physiological parameters. This research was carried out at the Rabbit Farming Unit of Pontifícia Universidade Católica do Paraná (PUCPR), −25.658030974669018, −49.28777735988852. All activities carried out in the laboratory must comply with the requirements set forth in: Law No. 6514, dated 22 September 1977, regarding occupational safety and health; Regulatory Standard No. 6 (NR-6), which regulates the use of Personal Protective Equipment (PPE); as well as the recommendations from ANVISA, RDC No. 306, dated 7 December 2004, which provides the Technical Regulation for the management of health service waste [28].

The protocol was approved by the Ethics Committee for the Use of Animals (CEUA) at PUCPR, under approval number 903—2nd version. The experiment started on 25 March 2019, and spanned 42 days. During this period, the ambient temperature at the site ranged from 17 °C (minimum) to 24 °C (maximum) in March. The average relative humidity was 65.6%. The photoperiod was set to 12 h of light and 12 h of darkness. Twenty-four New Zealand rabbits (both males and females) were used, each weaned at 35 days of age and having an initial average weight of 0.911 ± 0.130 kg. A randomized block design was used based on initial body weight, with animals housed individually in cages (80 × 60 × 45 cm) and allocated into three groups of eight rabbits per treatment (*n* = 8). Throughout the study period, the animals had unrestricted access to water and feed.

### 2.1. Nanofiber Production Process

**Figure 1:**
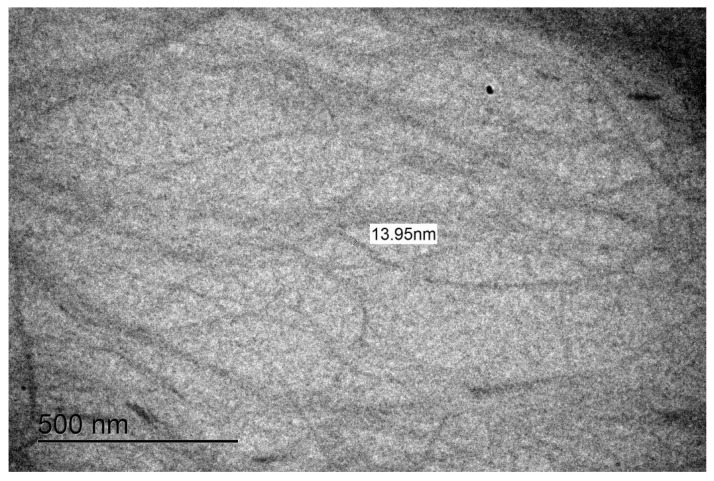
Development and Characterization of new nanofibers through Transmission Electron Microscopy (TEM).

The nanofibers, sourced from peach palm sheaths, were produced at the Wood Technology Laboratory of EMBRAPA Florestas in Colombo/PR using a mechanical processing method. The peach palm samples were fragmented using a 450 W blender for five minutes and then processed in a Super Masscolloider Masuko Sangyo microprocessor (Kawaguchi City, Japan). This equipment uses a rotating disk paired with a stationary disk, with an adjustable gap for material deposition. Processing parameters included 1500 rpm speed, 30 cycles, and a 0.1 mm disk gap to achieve the nanofiber gel, resulting in a 7% concentration of nanofiber gel [12] (Figure 1). After defibrillation, the gel containing 93% moisture was refrigerated and added to the experimental diets. The gel’s nanofiber composition featured NDF at 9.40%, ADF at 6.76%, mineral matter 0.01%, crude protein at 0.00%, ethereal extract at 0.17%, P at 1.45%, and Ca at 0.15% on a dry matter basis, according to the Chemical and Physical Methods for Food Analysis [29]. Diets were pelleted using an electric pellet machine (Grinder CAF-22 Stainless Steel 1/25 CV 300 Kg/h V17-M, Rio Claro, SP, Brazil) and dried in a forced-air oven (Fabbe, Primar—SL-102/1540, São Paulo, Brazil) at 55 °C for around 16 h to ensure suitable moisture levels for feed preservation.

### 2.2. Dietary Formulation

Based on nutritional standards for rabbit growth [30], the diets consisted of a control and two experimental treatments with a basal diet with 14% FB, without the inclusion of nanopupunha; a Diet 3.5%, diet with 3.5% inclusion of nanopupunha; and a Diet 10.5%, diet with 10.5% inclusion of nanopupunha. No performance-enhancing antibiotics were used in the diet. The ingredient composition and nutrient values are provided in Table 1.

### 2.3. Growth Performance Variables

In this study, we did not differentiate the results between males and females for the performance indicators, including final weight (FW), daily feed intake (DFI), and daily weight gain (DWG), which was measured by Weight gain = Final weight − Initial weight, and the feed conversion ratio (FC), which were determined from individual animal weight measurements at the beginning and end of the study. Feed intake was measured by weighing the feed provided, consumed, and wasted daily (Feed intake = Amount of feed offered − Amount of feed remaining). Diarrhea frequency was monitored daily using an adapted version [31] to observe the presence or absence of diarrhea [5].

### 2.4. Organ Morphometry

At the conclusion of the experiment, the rabbits were fasted for 12 h. Subsequently, all animals were humanely euthanized using a chemical method by a veterinarian, who administered pentobarbital at a dosage of 3 mg/kg of body weight, to collect samples from the jejunum and duodenum, cecal biological samples, and to measure the relative weights of their digestive organs (stomach, liver, small intestine—SI, and large intestine—LI) and accessory organs (kidneys and spleen) in a digital scale weighing (Marte AD3300, São Paulo/SP, Brazil). Organ weights were determined relative to the animals’ final body weight.

### 2.5. pH Measurements of Digestive Contents

Post-euthanasia, the digestive contents pH was measured in the stomach, jejunum, and cecum. Using a pH meter (Hanna Instruments, HI 99163, Smithfield, RI, USA), measurements were taken from specific regions: approximately 2 cm from the antropyloric region in the stomach, in the middle of the jejunum, and in the median area of the cecum, following a protocol adapted from [14,32].

### 2.6. Structural Intestinal Analysis

For structural analysis, 3 cm samples of the duodenum and jejunum were collected and stored in 10% formaldehyde for 72 h. At the PUCPR Histopathology Laboratory, samples were processed and embedded in paraffin. Four-micrometer sections were cut and stained using hematoxylin-eosin (HE). Samples were scanned using the Axio Scan Z1 microscope (ZEISS—Jena, Germany), and ZEISS software (ZEN 2.3) was used to perform the parameters such as villus height (AV), crypt depth (PC), villus width (LV), full mucosa thickness (ETM), and wall thickness (PE), along with the AV/PC ratio.

### 2.7. Ultrastructural Analysis of Intestinal Epithelium

Ultrastructural analysis was performed with samples 0.25 cm^2^ from the duodenum and jejunum, washed with saline solution (0.9%), and fixed in Karnovisk’s solution (2.5% glutaraldehyde and 3.424% cacodylate). Samples were then treated with osmium tetroxide, dehydrated, and dried in CO_2_ and were sputter-coated with gold and examined with a scanning electron microscope (Joel, JSM-6010PLUS/LA, software InTouchScope™, Tokyo, Japan) at Fundação Oswaldo Cruz (Fiocruz—Curitiba/PR, Brazil), focusing on villus structure and villus density (VD) within a 0.922 mm^2^ area, using six replications per treatment.

### 2.8. Blood Biochemical Parameters

Blood samples (5 mL) were collected on days 1 and 42 via cardiac vein puncture for analysis of blood glucose, total cholesterol, and triglycerides. Glucose levels were measured using the Accu-Chek Guide Kit (Basel, Switzerland), while cholesterol and triglyceride levels were assessed using Elitech kits (Sées, France). Plasma was obtained by centrifugation (10,000 rpm for 5 min) and stored at −20 °C for analysis with an EL80 automated reader at PUCPR’s Veterinary Hospital.

### 2.9. Immunological Parameters

Immunoglobulin levels were measured using Rabbit IgG and IgM ELISA Kits (ERB0171 and ERB0172, Fine Test, Wuhan, China). Blood samples were centrifuged, and serum was stored at −20 °C. Samples were diluted (IgG: 1:10,000; IgM: 1:30,000), and readings were taken at 450 nm using an Elisa reader (BioTek Instruments, Epoch2T, Winooski, VT, USA) [14].

### 2.10. Cecal Microbiota Analysis

Cecal content samples were collected in sterile tubes and stored on ice. For bacterial analysis, 1 g of digest was diluted in a 1% peptone water solution. Total Enterobacteriaceae and lactic acid bacteria were enumerated using MacConkey Agar and MRS Agar, respectively. Plates were incubated at 37 °C for 24–48 h, and bacterial counts were recorded according to FDA guidelines [33].

### 2.11. Statistical Analysis

All data were analyzed for model fit using Statgraphics^®^ 4.1. For performance parameters, initial body weight was used as a covariate. The model fit was assessed using the Generalized Linear Model (GLM) method, and the R^2^ values and the significance of regression coefficients evaluated the adequacy of the model. Data were analyzed using Type III ANOVA, with means compared by Tukey’s test and the LSD (Least Significant Difference) test. For each comparison of means, 95% LSD intervals were calculated, providing an estimate of the minimum significant differences between treatments. The assumptions of homoscedasticity and normality were verified using Levene’s test and the Shapiro–Wilk test, respectively. The *p*-values for these tests were <0.05. Additionally, scatter plots and histograms were generated for a visual assessment of homoscedasticity and normality assumptions. Regression was performed using the least squares method with individual values. Dunnett’s test was used for comparisons with the control, and a significance level of *p* < 0.05 was adopted for all analyses. All statistical analyses were conducted using SPSS 25 [34].

## 3. Results

### 3.1. Growth Performance Variables

There were no significant differences (*p* > 0.05) in the FBW, DFI, DWG, and FC of rabbits that received diets with different levels of nanopupunha (Table 2). No diarrhea was observed in any of the animals during the experimental period. The zootechnical difference between males and females was not considered, as it was not the study’s objective. Additionally, the number of animals used was low, respecting the principles of the 3Rs (Refinement, Replacement, and Reduction), aiming to minimize the use of animals without compromising the scientific validity of the study. Studies evaluating the performance of rabbits show that, by adopting appropriate practices and refined techniques, it is feasible to reduce the number of animals used in scientific research [35,36], while maintaining the integrity of the obtained results.

### 3.2. Organ Morphometry

The average organ morphometry of the growing rabbits is presented in Table 3.

**Figure 2:**
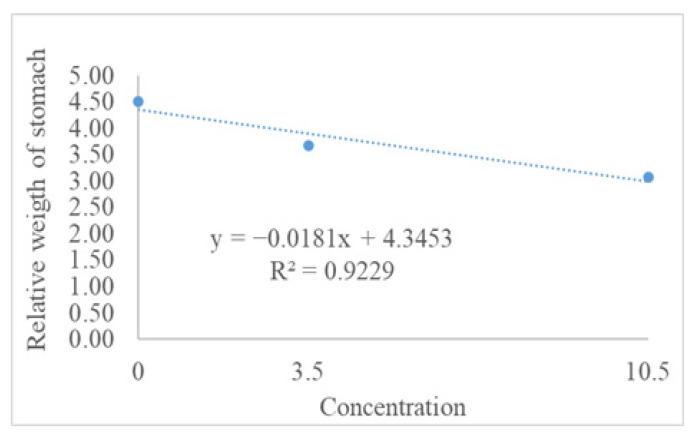
Decreasing linear effect of nanopupunha inclusion levels added to the diet on the relative stomach weight of growing rabbits.

**Figure 3:**
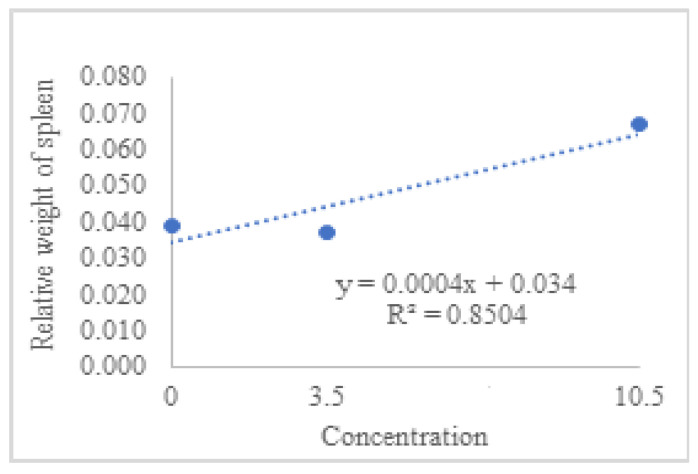
Increasing linear effect of nanopupunha inclusion levels added to the diet on the relative spleen weight of growing rabbits.

The inclusion of nanopupunha showed a decreasing linear effect (R^2^ = 0.922) on relative stomach weight and an increasing linear effect (R^2^ = 0.850) on relative spleen weight, Figure 2 and Figure 3, respectively. In quantitative terms, for each 1% addition of nanopupunha, the weight of the stomach decreased by 0.180%, whereas that of the spleen increased by 0.0004%.

### 3.3. pH Measurements of Digestive Contents

There were no significant differences in digestive tract pH (Table 4).

### 3.4. Structural and Ultrastructural Analysis of the Intestinal Epithelium

The averages of the structural and ultrastructural analyses of the intestinal epithelium of growing rabbits are presented in Table 5.

**Figure 6:**
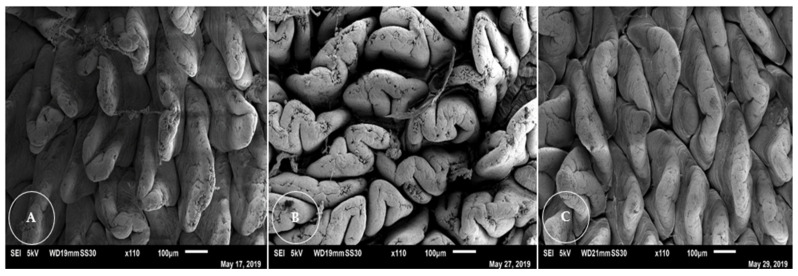
Ultrastructural analysis of the duodenum of rabbits that received the diets: Control—0% Nanofibers—(**A**), 3.5% nanopupunha—(**B**), and 10.5% nanopupunha—(**C**).

**Figure 7:**
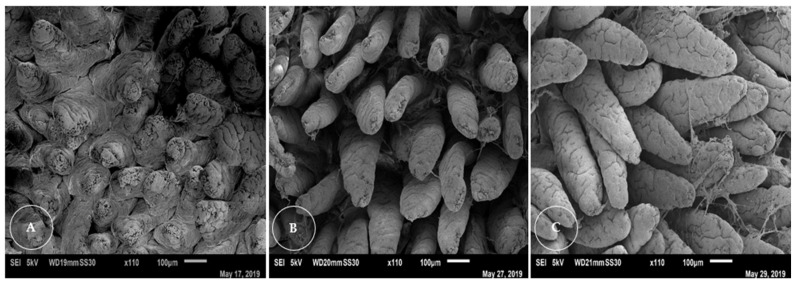
Ultrastructural analysis of the jejunum of rabbits that received the diets: Control—0% Nanofibers—(**A**), 3.5% nanopupunha—(**B**), and 10.5% nanopupunha—(**C**).

Ultrastructural analysis of the duodenum and jejunum, Figure 6 and Figure 7, respectively, revealed that the villi of the duodenum were in the shape of flatter plaques in the Control and Diet 3.5% treatments and in the shape of a tongue in the Diet 10.5% treatment, without evidence of cell loss, either in the duodenum or jejunum. The jejunal villi were thin and finger-shaped. However, rabbits that received the 3.5% and 10.5% nanopupunha diets presented more intact villi and epithelial cells than the control group.

### 3.5. Blood Biochemical Parameters

The average glycemia levels of the rabbits did not change (*p* > 0.05) from the 35th to the 77th day (Table 6).

**Figure 8:**
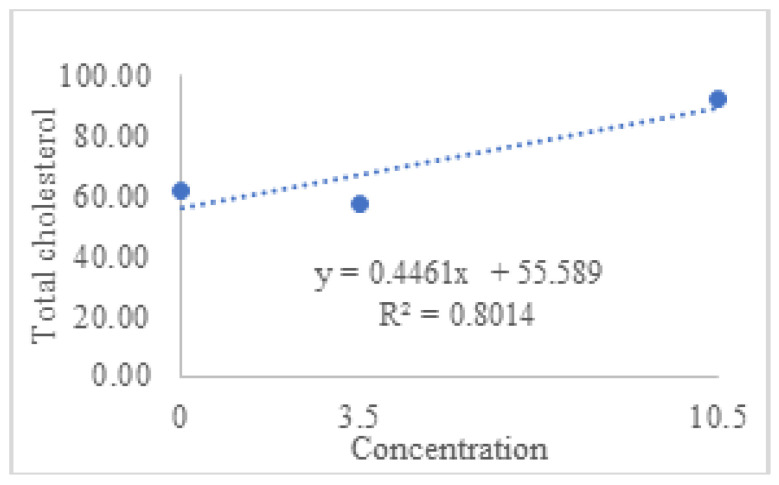
Increasing linear effect of nanopupunha inclusion levels added to the diet on total cholesterol in growing rabbits.

There was an increasing linear effect of nanopupunha inclusion levels on the total cholesterol rate (R^2^ = 0.801), with each 1% addition of nanopupunha increasing total cholesterol by 0.446 mg/dL, Figure 8.

### 3.6. Immunological Parameters

**Figure 9:**
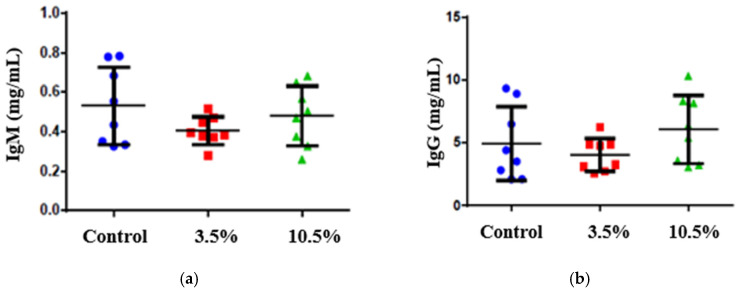
Serological results by ELISA-IgM (**a**) and ELISA-IgG (**b**) tests in blood serum samples from rabbits fed the Control, 3.5% nanopupunha, and 10.5% nanopupunha diets.

The levels of serum IgM and IgG did not change significantly (*p* > 0.05) among the treatments in rabbits fed different levels of dietary nanopupunha, Figure 9a,b.

### 3.7. Cecal Microbiota Analysis

There was no difference (*p* > 0.05) in the counts of Enterobacteriaceae and lactic acid bacteria in the cecal contents of growing rabbits fed different levels of nanopupunha added to the diet (Table 7).

## 4. Discussion

### 4.1. Growth Performance

The final live weights of the rabbits that consumed diets with 3.5% and 10.5% nanopupunha were 2.284 and 2.246 kg, respectively, and did not differ statistically from those that received the control treatment (2.228 kg). However, it was higher than the weights proposed by [37], who observed an average final live weight of 2.090 kg for New Zealand rabbits at 75 days of age. The higher final live weight in the present study indicates that the processing method and inclusion of peach palm nanofibers in the diet are safe, providing satisfactory performance for growing rabbits.

Nanotechnology can aid in the development of new products for animal nutrition, thereby increasing the efficiency of dietary nutrient utilization [11] reported an increase in DWG (*p* < 0.05) in piglets fed chitosan nanoparticles. They reported that nanoparticles have higher bioavailability and promote greater benefits to animal performance by improving the utilization of diet nutrients. Nanofibers have greater digestion and absorption capacity than fibers owing to their higher bioavailability, biocompatibility, and hydrogen bonds, which increase their reactivity [38,39].

### 4.2. Organ Morphometry

During the growth phase, the development of healthy organs and the digestive tract are determining factors in the performance of rabbits and are related to the fibrous composition of the diet [40,41]. Studies have observed that fibers directly affect the size and weight of organs in birds [11], and differences in organ weights are related to the chemical nature of fibers [42,43], with primary effects on the functions of the digestive tract [44] and on the development of the animals’ digestive tract. It is well established that dietary fiber, particularly fibers with specific characteristics such as pupunha nanofibers, can influence the growth and morphometry of digestive organs. Fiber has a direct impact on intestinal motility and the composition of the gut microbiota, factors that can, in turn, affect the size and function of organs such as the stomach and spleen. Although the observed percentage variations are small (the 0.0004% increase in spleen weight), it is important to consider that these changes may be the result of physiological adaptations associated with the action of nanofibers in the gastrointestinal tract. Additionally, the individual genetic characteristics of the rabbits can also influence organ development. The interaction between dietary fiber and these genetic characteristics can lead to variations in the morphometry of the organs observed in rabbits.

In the present study, the inclusion of nanopupunha added to the diet resulted in a linear decrease in the relative weight of the stomachs of growing rabbits. Previous studies have described that insoluble NSPs increase the rate of passage of the diet through the digestive tract [45], causing greater emptying of the digestive tract [14]. Peach palm nanofibers have a high content of insoluble NSPs, which may contribute to an increased rate of passage through the diet, resulting in a lower full stomach weight. In addition, the fiber content of the ingredients and the size of the fibrous particles favor the development of the monogastric digestive tract [46].

Conversely, the inclusion of nanopupunha added to the diet resulted in a linear increase in the relative spleen weight of rabbits. According to [47], changes in the spleen can be induced by toxic substances and antinutritional factors. An appropriate nutritional balance of NSPs can minimize the incidence of diseases, improve organ development, increase the expression of immune-related genes, and promote the maturation of defense cells [48]. Dietary insoluble NSPs can favor beneficial microbial populations in the large intestine [49] and improve the synthesis of short-chain fatty acids (SCFAs), which are fundamental for the maturation of the immune system [50]. These factors increase the proliferation of immune cells in the intestine and spleen [51,52], stimulating a non-specific immune response and slightly increasing spleen weight [53,54], with positive effects on animal health [55]. Thus, the interaction of dietary bioactive feed components with the intestinal tract and organs of the immune system improves the immune response and reduces susceptibility to intestinal and metabolic diseases.

### 4.3. Structural Analysis of the Intestinal Epithelium

Maintaining intestinal health in growing rabbits is essential for optimizing productivity, as balanced intestinal function promotes the development of healthy villi with increased villus height and mucin production [56]. These factors provide better protection for the intestinal epithelium and enhance nutrient utilization. In this study, the inclusion of nanopupunha in the diet had a linear effect on duodenal crypt depth, a finding consistent with the results of [57], who observed a similar effect with the inclusion of insoluble non-starch polysaccharides (NSPs) in the diet of rabbits. Additionally, ref. [58] reported a linear increase in both crypt depth and villus height with the inclusion of insoluble NSPs in growing rabbits’ diets. This pattern was also observed in the present study, suggesting that pupunha nanofibers may play a role similar to other insoluble fibers, promoting beneficial intestinal adaptations.

The inclusion of nanopupunha in the diet also resulted in a linear increase in total jejunal mucosa thickness, an effect that may be related to the structural properties of insoluble fibers. Studies indicate that the chemical composition of insoluble fibers directly influences the thickness of the jejunal muscular layer [59,60], and our findings reinforce this relationship. Indeed, previous research suggests that digestible NSPs are strongly associated with mucosal integrity, digestibility, and nutrient absorption capacity, positively impacting the intestinal health of monogastric animals [14,58]. The intestinal response observed in this study, particularly the increased crypt depth and preserved villus integrity, suggests that nanopupunha may act as a positive modulator of intestinal morphology, possibly stimulating cell renewal and enhancing epithelial barrier function. The preservation of villus structure, particularly with the inclusion of 10.5% nanopupunha, reinforces the hypothesis that this ingredient may contribute to improved rabbit intestinal health. These findings align with those of [61], who reported that dietary insoluble fibers enhance the integrity and functionality of the intestinal mucosa in growing rabbits.

Beyond its structural effects, nanopupunha may influence other physiological mechanisms involved in intestinal health. Research suggests that plant-based nanofibers may positively impact the gut microbiota by stimulating the production of short-chain fatty acids (SCFAs), which play a fundamental role in maintaining intestinal homeostasis and modulating inflammatory responses [62]. This effect could contribute to improved nutrient absorption and small intestine barrier function while reducing the entry of pathogenic antigens. Given these results, the inclusion of nanopupunha in the diet appears to be a promising approach to enhancing intestinal health and productivity in rabbits. However, further studies are needed to deepen our understanding of the digestibility mechanisms of nanopupunha NSPs and their impact on gut microbiota and animal metabolism. Additionally, comparative investigations with other sources of insoluble fiber could help determine the specific efficacy of nanopupunha in rabbit nutrition.

### 4.4. Blood and Immunological Biochemical Parameters

Natural biopolymer derivatives, such as cellulose at the nanoscale, have various food applications and the potential to enhance nutrient absorption (glucose and lipids), with significant implications for the homeostatic balance of the organism [13]. Nanofibers can alter glucose and lipid absorption in the gastrointestinal tract [10]. According to [63,64], the fasting glycemic range in rabbits varies from 75 to 155 mg/dL. These values are lower than those found in this study, which were 154.37 mg/dL and 164.87 mg/dL for 3.5% and 10.5% nanopupunha in the diet, respectively. Elevated glucose levels may indicate an alteration in glucose absorption or a modulation of the insulin response. Non-starch polysaccharides (NSPs) play a crucial role in maintaining homeostasis in rabbits by regulating glucose digestion and absorption [65] and stabilizing blood glucose levels. Additionally, fiber-induced modulation of the intestinal microbiota can impact carbohydrate metabolism, influencing blood glucose levels [66]. Therefore, the inclusion of nanopupunha may affect glycemic metabolism in growing rabbits.

We observed a linear increase in total cholesterol levels with the inclusion of nanopupunha in the diet of growing rabbits. Total cholesterol levels ranged from 57.12 mg/dL (3.5% nanopupunha) to 92.25 mg/dL (10.5% nanopupunha), which are higher than the values reported by [56], ranging from 58.40 to 68.31 mg/dL. The increase in total cholesterol observed with nanopupunha supplementation may be attributed to greater energy absorption from the diet and enhanced synthesis of high-density lipoprotein (HDL), a well-recognized benefit of dietary fiber [65]. Non-starch polysaccharides (NSPs) improve digestion, intestinal transit [13], and dietary lipid utilization in monogastric animals [67], which may contribute to increased circulating cholesterol levels. It is important to consider that different fiber types and their physicochemical properties can exert distinct effects on lipid metabolism [68]. According to [19], cellulose nanofibers can reduce triglyceride hydrolysis and absorption in the small intestine. The inclusion of nanopupunha in the diet of growing rabbits has the potential to positively influence digestion, nutrient absorption, and intestinal health. Further studies are recommended to elucidate the mechanisms by which nanopupunha affects energy and lipid metabolism, as well as to assess the long-term implications for rabbit health.

Immunological and intestinal pathologies, including intestinal inflammation, cancer, and autoimmune diseases, account for approximately 70% of all diseases in rabbits [69] and are associated with the intestinal microbiota profile, which, in turn, can be modulated by fiber intake [70,71]. Additionally, dietary fiber consumption promotes beneficial microbiota, which can enhance gut barrier function and modulate immune cell activity [72]. In this study, serum IgG and IgM levels were statistically similar among the different rabbit groups. However, the values obtained were higher than those reported by [73]. According to [45], nutritional factors modulate immune responses in the intestines of growing rabbits, and fiber intake promotes microbiota diversity [70] and enhances the synthesis of short-chain fatty acids (SCFAs) [40], which are essential for immune system maturation [45]. Furthermore, SCFAs produced through the fermentative degradation of dietary fiber in the intestine may reduce the interaction of potentially pathogenic agents with epithelial cells [72,73,74], potentially improving nutrient utilization from the diet. Therefore, incorporating nanopupunha into the diet of growing rabbits may have positive effects on immune function and infection resistance, contributing to a healthier digestive system. The use of nanopupunha as a dietary ingredient for animals may thus offer an innovative approach to promoting intestinal and immune health.

### 4.5. Cecal Microbiota Analysis

Dietary fiber can modulate the commensal microbiota by modifying the fermenting microbial populations and SCFA synthesis in the large intestine [41,71], altering the concentrations of acetic, propionic, and butyric acids in the cecum of rabbits [57], and providing greater energy input to animals, resulting in enhanced growth and greater productive gains. However, there was no significant difference in the counts of enterobacteria and lactic acid bacteria in the large intestine of rabbits fed dietary nanopupunha.

## 5. Conclusions

Overall, 10.5% of nanopupunha provided the quality and quantity of fiber needed to improve the development and health of the digestive tract of rabbits. Nanotechnology can contribute to improving the quality of nutrition for rabbits, in addition to increasing the use of co-products from agricultural industries, making important contributions to animal nutrition.

## Figures and Tables

**Figure 4:**
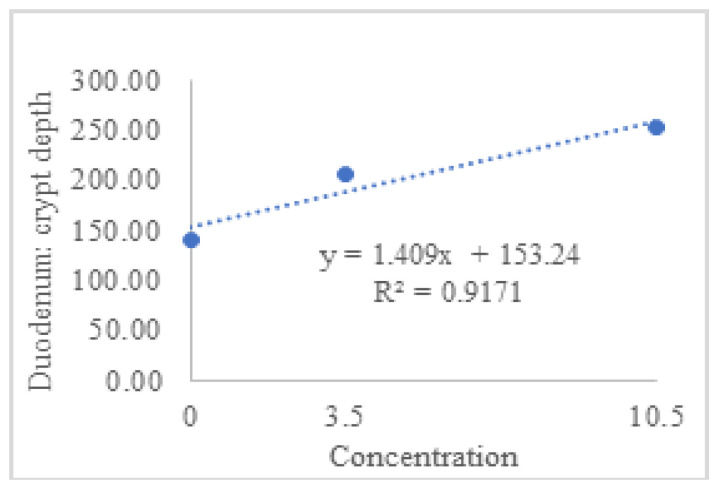
Increasing linear effect of nanopupunha inclusion levels added to the diet on the duodenal crypt depth of growing rabbits.

**Figure 5:**
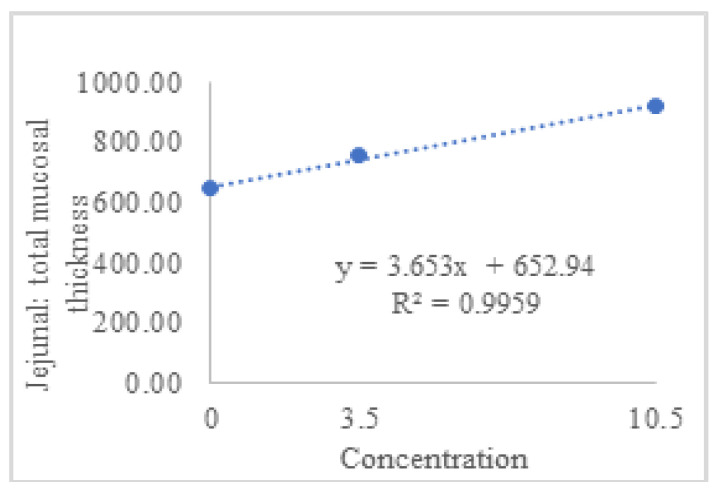
Increasing linear effect of nanopupunha inclusion levels added to the diet on the jejunal total mucosal thickness of growing rabbits.

**Table 1:** Composition and nutritional values of the calculated and analyzed diets for growing rabbits.

Ingredients %	Nanopupunha		
	Control	3.5%	10.5%
Corn, 8% CP	22.50	29.21	35.21
Wheat bran, 16% CP	31.50	28.00	16.00
Soybean meal, 45% CP	26.00	25.00	28.00
Cellulose	10.00	6.20	0.00
Nanopupunha	0.00	5.00	13.50
L-Cystine	0.25	0.25	0.25
Cristal Sugar	3.00	2.30	3.00
Molasses aroma	0.03	0.03	0.03
^2^ SINOX PLUS	0.01	0.01	0.01
^1^ Premix Nuvilab coelhos	4.00	4.00	4.00
Total	100.00	100.00	100.00
Calculated nutritional values			
Dry matter, %	90.13	90.35	90.72
Crude protein, %	18.53	18.10	18.01
Ethereal extract, %	2.56	2.66	2.43
Crude Fiber, %	14.00	14.00	14.00
Nanopupunha, %	0.00	3.50	10.50
Grude energy, (Kcal/Kg)	3.304	3.385	3.306
L-lysine, %	0.10	0.10	0.10
DL-methionine, %	0.31	0.31	0.31
Mineral matter	6.70	6.71	6.31
Total phosphorus, %	0.63	0.64	0.60
Calcium, %	0.98	0.97	0.94
Nutritional values analyzed			
NDF, %	28.50	22.50	21.60
ADF, %	15.40	11.40	9.15

Crude protein (CP); acid detergent fiber (ADF); neutral detergent fiber (NDF). ^1^ Composition of vitamin and mineral supplement per kg of feed: vit A 8000 uI/kg, vit D3 1200 uI/kg, vit E 20 uI/kg, vit K3 1 mg/kg, vit B1 2 mg/kg, vit B2 6 mg/kg, vit B6 2 mg/kg, vit B12 10 mcg/kg, Nacin 30 mg/kg, pantoten calcium 17 mg/kg, Folic acid 1 mg/kg, Biotin 0.03 mg/kg, Choline 1400 mg/kg, Minerals: Na 2700 mg/kg, Fe 40 mg/kg, Mn 40 mg/kg, Zn 60 mg/kg, Cu 6 mg/kg, I 0.3 mg/kg, Se 0.1 mg/kg, Co 1 mg/kg, F 60 mg/kg, BHT 100 mg/kg. ^2^ SINOX PLUS.

**Table 2:** Growth performance of growing rabbits fed with different levels of nanopupunha added to the diet.

Variables ^1^		Nanopupunha		SEM ^2^	*p*-Value	Regression
	Control	3.5%	10.5%			R^2^
IBW, kg	0.92	0.91	0.91	0.13	0.985	0.38
FBW, kg	2.22	2.28	2.24	0.04	0.924	0.01
DFI, kg	0.11	0.11	0.12	0.02	0.215	0.47
DWG, kg	0.03	0.03	0.03	0.08	0.526	0.02
FC	3.75	3.41	3.94	0.10	0.150	0.26

^1^ Averages of the initial body weight (IBW), final body weight (FBW), daily feed intake (DFI), daily weight gain (DWG), and feed conversion (FC). ^2^ SEM: standard error of the mean.

**Table 3:** Means of organ morphometry of the digestive tract and adnexal organs of growing rabbits fed with different levels of nanopupunha added to the diet.

Variables ^1^	Nanopupunha			SEM ^2^	*p*-Value	Regression
	Control	3.5%	10.5%			R^2^
FBW, kg (77 d)	2.22	2.28	2.24	0.04	0.924	0.48
^3^ Stomach, %	4.49	3.66	3.06	0.24	0.043	0.92
SI, %	2.41	2.28	2.69	0.07	0.065	0.64
LI, %	9.98	9.42	10.38	0.28	0.723	0.33
Liver, %	3.52	4.19	3.91	0.16	0.535	0.18
Kidneys, %	0.73	0.69	0.78	0.02	0.293	0.43
^3^ Spleen, %	0.04	0.04	0.08	0.00	0.001	0.85

Final Body Weight (FBW). ^1^ Average weights of stomach, small intestine (SI), large intestine (LI), liver, kidneys, and spleen, depending on treatment. ^2^ SEM—standard error of the mean. ^3^ Linear effect of nanopupunha inclusion levels in the diet of growing rabbits (*p* < 0.05).

**Table 4:** Mean pH values of the contents of the filled digestive tract of growing rabbits fed with different levels of nanopupunha added to the diet.

Variables	Nanopupunha			SEM	*p*-Value	Regression
	Control	3.5%	10.5%			R^2^
Stomach pH	2.27	2.47	2.08	0.10	0.342	0.41
Jejunum pH	6.96	6.93	7.00	0.06	0.733	0.55
Cecum pH	6.48	6.58	6.51	0.06	0.947	0.08

SEM—standard error of the mean.

**Table 5:** Structural and ultrastructural analysis of the intestinal epithelium of growing rabbits, fed with different levels of nanopupunha added to the diet.

Varibles ^1^	Nanopupunha			SEM ^2^	*p*-Value	Regression
	Control	3.5%	10.5%			R^2^
Duodenum						
VH (µm)	681.10	833.95	664.75	37.82	0.477	0.75
^3^ CD (µm)	141.00	206.80	252.79	7.50	0.028	0.91
VH:CD	4.39	4.22	3.30	0.39	0.378	0.96
VD *	29.77	32.58	31.99	1.64	0.679	0.37
VW (µm)	115.08	123.15	123.16	4.85	0.621	0.57
TMT (µm)	1153.62	1219.98	1326.04	44.90	0.194	0.99
WT (µm)	86.80	122.24	107.67	7.50	0.493	0.17
Jejunum						
VH (µm)	533.46	579.14	666.15	47.67	0.331	0.99
CD (µm)	116.61	123.92	130.00	7.80	0.560	0.94
VH:CD	4.22	4.93	4.49	0.54	0.508	0.03
VD *	32.08	27.94	32.58	2.82	0.845	0.08
VW (µm)	117.68	125.60	139.24	6.25	0.236	0.99
^3^ TMT (µm)	646.14	754.46	923.51	56.43	0.034	0.99
WT (µm)	87.45	114.53	71.62	7.22	0.332	0.28

^1^ Villus height (VH), crypt depth (CD), VH: CD ratio, villus density (VD), villus width (VW), total mucosal thickness (TMT), and wall thickness (WT) of the duodenum and jejunum of growing rabbits; ^2^ SEM, standard error of the mean. ^3^ Linear effect of nanopupunha inclusion levels in the diet of growing rabbits (*p* < 0.05). VD *—number of villi/922 μm^2^. The inclusion of nanopupunha showed an increasing linear effect for CD of the duodenum (R^2^ = 0.917), with each 1% addition of nanopupunha increasing the crypt depth by 1.409 µm, Figure 4. Dietary nanopupunha showed an increasing linear effect (R^2^ = 0.995) on TMT, with each 1% addition of nanopupunha increasing the total thickness of the jejunal mucosa by 3.653 µm, Figure 5.

**Table 6:** Average glycemia levels of rabbits at (35 days old) and levels of glucose, cholesterol, and triglycerides (mg/dL) at 77 days old.

Variables (mg/dL)	Nanopupunha			SEM ^1^	*p*-Value	Regression
	Control	3.5%	10.5%			R^2^
35 days old						
Glycemia	181.375	138.000	152.875	12.391	0.369	0.240
77 days old						
Glycemia	159.250	154.375	164.875	9.254	0.780	0.469
^2^ Cholesterol	62.000	57.125	92.250	6.618	0.039	0.801
Triglycerídes	75.500	71.125	92.000	5.518	0.162	0.741

^1^ SEM—standard error of the mean. ^2^ Linear effect of inclusion levels of nanopupunha added to the diet of growing rabbits (*p* < 0.05).

**Table 7:** Enterobacteriaceae and lactic acid bacteria count in the cecal content of rabbits fed diets containing different levels of Nanopupunha.

Log CFU/g	Nanopupunha			SEM ^1^	*p*-Value	Regression
	Control	3.5%	10.5%			R^2^
Enterobacterias sp	8.997	8.299	7.899	0.231	0.060	0.885
Lactic acid	6.298	6.485	5.845	0.279	0.464	0.660

^1^ SEM: Standard error of the mean.

## Data Availability

The data supporting this study will be made available upon request.
